# Effect of Pyrochar and Hydrochar on Water Evaporation in Clayey Soil under Greenhouse Cultivation

**DOI:** 10.3390/ijerph16142580

**Published:** 2019-07-19

**Authors:** Yang Liu, Xiaoyu Liu, Ni Ren, Yanfang Feng, Lihong Xue, Linzhang Yang

**Affiliations:** 1 Research Center of IoT Agriculture Applications/Institute of Agricultural Information, Jiangsu Academy of Agricultural Sciences, Nanjing 210014, China; 2 Key Laboratory of Agro-Environment in Downstream of Yangtze Plain, Ministry of Agriculture and Rural Affairs/Institute of Agricultural Resources and Environment, Jiangsu Academy of Agricultural Sciences, Nanjing 210014, China; 3 Jiangsu Vocational College of Agriculture and Forestry, Jurong 212400, China; 4 Stockbridge School of Agriculture, University of Massachusetts, Amherst, MA 01003, USA; 5 School of the Environment and Safety Engineering, Jiangsu University, Zhenjiang 212001, China

**Keywords:** soil evaporation, hydrochar, pyrochar, greenhouse cultivation, clayey soil

## Abstract

Greenhouse cultivation consumes large volumes of freshwater, and excessive irrigation induces environmental problems, such as nutrient leaching and secondary salinization. Pyrochar (biochar from high-temperature pyrolysis) is an effective soil amendment, and researches have shown that pyrochar application could maintain soil nutrient and enhance carbon sequestration. In addition to pyrochar from pyrolysis, hydrochar from hydrothermic carbonization is considered as a new type of biochar and has the advantages of low energy consumption and a high productive rate. However, the effect of these two biochars on water evaporation in clayey soils under a greenhouse system has seldom been studied. The relationship between water evaporation and biochar properties is still unknown. Thus, in the present study, water evaporation under pyrochar and hydrochar application were recorded. Results showed that both pyrochar and hydrochar application could inhibit water evaporation in clayey soil under greenhouse cultivation. Pyrochar showed a better inhibition effect compared with hydrochar. Correlation analysis indicated that the water evaporation rate was significantly positively correlated with bulk density of biochar (*p* < 0.05). Overall, application of pyrochar or hydrochar could both reduce soil bulk density and inhibit soil evaporation, and be available for greenhouse cultivation. However, the inhibition effect depends on the properties of the biochar.

## 1. Introduction

Greenhouse cultivation has developed rapidly in recent years in China [1], and by 2017, the area under greenhouse cultivation reached 3.7 Mha [2]. Due to the enclosed environment, greenhouse cultivation cannot utilize natural precipitation. For optimal profit, large volumes of freshwater are consumed as irrigation. However, large areas of China have the problem of water shortage [3] and the water resource per capita is only one-fourth of the world average [4]. Furthermore, excessive irrigation induces the risk of nutrient loss and secondary soil salinization [2]. Soil evaporation (ES) is a major proportion of water loss in agriculture worldwide, and 30% to 75% of the growing season’s rainfall is lost by ES [5]. Therefore, improvement in greenhouse cultivation water use with the view to reduce ES is important for practical cultivation and environmental protection.

Recently, biochar has been used as an effective soil amendment for the improvement of nutrient retention [6,7], soil carbon sequestration [8], and pollution remediation [9,10]. Biochar can also reduce CO_2_, CH_4_, and N_2_O production in soil [11]. Generally, there are two types of biochar based on different production processes. Pyrochar is a stable, recalcitrant organic carbon compound, produced when biomass is heated to temperatures, usually between 300 and 700 °C, under low oxygen concentrations [12]. Hydrochar is a new type of biochar produced by hydrothermic carbonization (HTC), compared with pyrochar by pyrolysis [13]. HTC is a low-temperature transformation process (temperatures between 180 and 300 °C) performed in the presence of water and high pressure in an oxygen-free environment [14]. Hydrochar has the advantages of low energy consumption, being environmental-friendly during production, and a high productive rate [15]. Therefore, hydrochar has recently been receiving increasing attention [16].

Researchers have conducted experiments to study the effects of pyrochar application on soil water content, and the results have shown that pyrochar addition could increase the soil water-holding capacity [17]. Due to the high specific surface area (SSA) and porosity [18], pyrochar application inevitably influences the physical and chemical properties of soils and further influence the ES.

The effect of pyrochar application on ES has been investigated. Xu et al. (2016) studied the effect of pyrochar from different feedstocks with different particle size on ES [19]. Results showed that effect of pyrochar on ES was depended on feedstock, particle size, and application proportion of pyrochar. Results from Wang et al. (2018) showed that pyrochar application enhanced soil water retention and inhibited ES, and ES decreased as the application proportion and particle size of pyrochar [20] increased. Researches also showed that the effect of pyrochar application on ES depended on the properties of the pyrochar. For example, pyrochar with a larger pore volume and average pore diameter had better water retention capacity [21]. Most researches indicated that pyrochar amendments could improve soil water retention [22] and irrigation water productivity [23]. However, researches on the effect of pyrochar application on ES were mainly focused on sandy soil in arid regions [24]. Until now, researches have seldom been conducted in fine-textured soil [25], e.g., clayey soil in greenhouse cultivation. Clayey soil always has a high bulk density, low permeability, and the mechanism of soil water movement is inevitably different from sandy soil [26]. To our knowledge, the effect of biochar application on ES in clayey soil has not been reported. Furthermore, patterns and mechanisms of different biochars, especially hydrochar, on ES are still unclear. Thus, in the present study, the effect of pyrochar and hydrochar with different application proportions on ES was investigated. The present study could offer a comprehensive evaluation of the effect of pyrochar and hydrochar on ES in clayey soil and a possible alternative method for saving irrigation in greenhouse cultivation.

## 2. Materials and Methods 

### 2.1. Materials

Clayey soil used in the experiment was sampled from the surface layer (0–20 cm) of a strawberry greenhouse in Nanjing City, Jiangsu Province (32°00′14.3″ N, 119°04′36.5″ E). Soil carbon content was 8.72 g/kg, saturation capacity was 42%, and sand, silt, and clay contents were 20.5%, 26.2%, and 53.3%, respectively. After sampling, clayey clods were broken up with a rubber pestle, and then soils were dried at room temperature before being passed through a 2 mm sieve. In total, 4 types of pyrochar (SP500, SP700, WP500, and WP700, which were sawdust and wheat straw pyrolysis at 500 and 700 °C, respectively. SP and WP represent sawdust pyrochar and wheat straw pyrochar.) and 2 types of hydrochar (SH260 and WH260, which were sawdust and wheat straw by HTC at 260 °C, respectively. SH and WH represent sawdust hydrochar and wheat straw hydrochar.) were used in the present experiment. All 6 types of biochar were produced in Jiangsu Academy of Agricultural Sciences. The basic properties are shown in Table 1.

### 2.2. Experimental Design and Analysis

In total, 12 biochar application treatments were established (6 types of biochar in Table 1 at proportions of 2% and 6% *w*/*w*, named as SP500-2%, etc.) with no biochar applied as control (CK). Each treatment had 3 replicates. An aluminum column with 5.4 cm diameter and 3.2 cm height was used to conduct the experiment. Forty grams of soil with a relative proportion of biochar was completely mixed before being filled into columns. Twenty milliliters of distilled water (over the saturation capacity 42%) was added in each column. All the columns were incubated in the laboratory with a controlled temperature at 25 °C (normal average temperature under a greenhouse system). The weight of each column was recorded every 12 h, and the weight difference between two observation was calculated as water loss by ES. The observation was continued until the weight difference between two observations was less than 0.2 g. After incubation, columns were heated at 105 °C to a constant weight. The volume of soil in each column was recorded to calculate soil bulk density (BDs). 

The time when water loss reached the ES points of 5, 10, and 15 g (25%, 50%, and 75% of the total water) were calculated based on the observations. The average ES rate (%/h) when water loss reached 5, 10, and 15 g (25%, 50%, and 75% of the total water, namely R25, R50, and R75) was calculated by the ratio of water loss (25%, 50%, and 75%) divide the time when it was reached.

### 2.3. Statistical Analysis

One-way analysis of variance (ANOVA) and multivariate analysis was conducted to determine significant differences between the treatments in the incubation experiment. The least significant difference (LSD) between means was estimated using *p* < 0.05 as the standard for significance. A first-order kinetics equation was used to fit the curves of water loss (ES). Correlation analysis was conducted between biochar properties and ES. Statistical analysis was performed using the SPSS 19.0 (SPSS Inc., Chicago, IL, USA) for Windows software package. 

## 3. Results

### 3.1. Soil Evaporation (ES)

**Figure 1:**
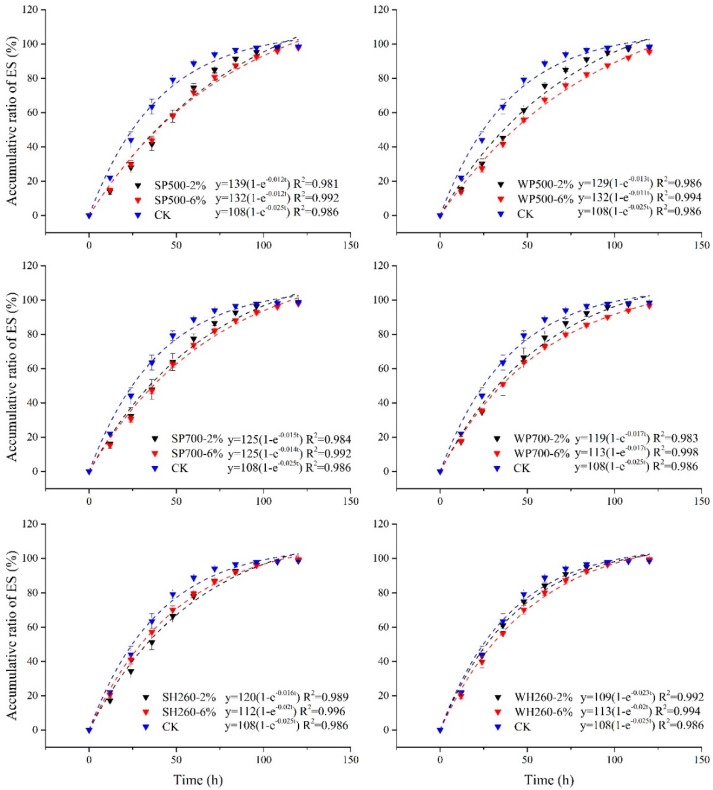
Accumulative soil evaporation and its first-order kinetics equation fitting under pyrochar and hydrochar application. Error bars represent standard deviations.

Our observations showed that ES reached a high rate at the beginning and declined gradually to a new balance (Figure 1). Biochar application reduced the ES rate compared with CK. During the midterm of observation (36–84 h), the ES rate of both pyrochar and hydrochar application was significantly lower than CK. And pyrochar application showed a better ES inhibition than hydrochar (Figure 1). Moreover, a higher pyrochar application proportion showed a better inhibition on ES. In most observation, an application proportion at 6% showed a lower cumulative water loss than that at 2%. But in contrast, hydrochar application at 2% and 6% showed a similar trend (Figure 1). 

**Figure 2:**
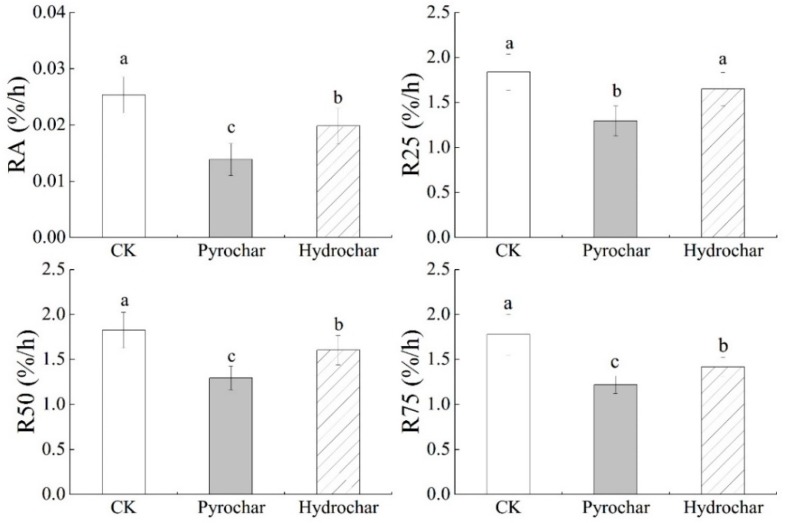
Soil evaporation rate at different times under pyrochar and hydrochar treatments. RA represents the average soil evaporation (ES) rate. R25, R50, and R75 represent ES rate when water loss reached 25%, 50%, and 75% of the total water. Different lowercase letters represent statistical differences at *p* < 0.05. Error bars represent standard deviations.

By comparison of several equations (Table 2), a first-order kinetics equation was used for fitting ES curves under different biochar application treatments and showed good fitting results (*p* < 0.01). Parameter b in first-order kinetics equation was used to represent the average ES rate (RA). The results showed that RA was highest under CK. RA under pyrochar application treatment was lower than that under hydrochar application, which further indicated a better ES inhibition of pyrochar (Figure 2).

*y* = a*t* + b, *y* = N_0_ (1 − e^−b*t*^) for linear and first-order kinetics equation, respectively. *t* and N_0_ represent time and parameter of first-order kinetics equation, respectively.

### 3.2. Soil Bulk Density (BDs)

**Figure 3:**
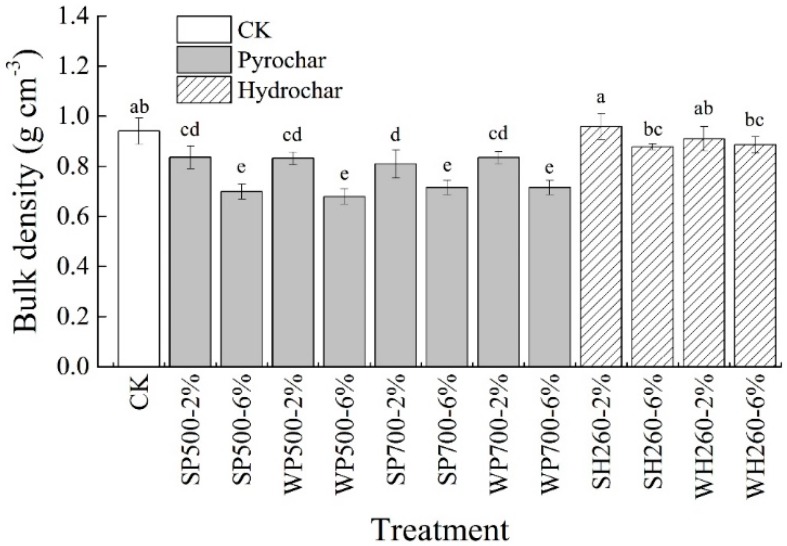
Soil bulk density under different pyrochar and hydrochar treatments after the incubation experiment. Different lowercase letters represent statistical differences at *p* < 0.05. Error bars represent standard deviations.

Clayey soil is always heavy textured with large bulk density (BDs). In the present study, both pyrochar and hydrochar application reduced BDs (Figure 3). The average BDs was reduced by 8.2% and 18.9% under application proportion at 2% and 6% compared with CK, respectively. The result was mainly due to the low BDc of pyrochar and hydrochar. In the present study, BDc of pyrochar was significantly lower than hydrochar (0.11 and 0.31 g/cm^3^ for pyrochar and hydrochar, respectively). Thus, pyrochar showed a better bulk density reduction compared with hydrochar. A higher application proportion increased BDs reduction. At 2% and 6% application proportion, BDs reduced by 11.9%, 0.7% and 25.3%, 6.2% under pyrochar and hydrochar application, respectively (Figure 3). However, feedstock and prepared temperature showed no effect on BDs. Results of the present study showed that biochar application, especially pyrochar application, reduced BDs, and further improved soil structure and affected ES of clayey soils.

## 4. Discussion

### 4.1. Biochar Properties on Soil Evaporation

Pyrochar application inhibited ES. The average ES rate (RA) under pyrochar application reduced by 45.3% compared with that under CK (Figure 2). The results indicated that pyrochar application improved soil water retention, which was in agreement with former researches [20,21]. Furthermore, hydrochar application also inhibited ES compared with CK (RA reduced by 21.8%), which is lower than the pyrochar application. Properties of these two types of biochar in the present study showed significant differences (Table 1), which further affected ES. Therefore, ES inhibition was quite different for these two types of biochar.

Results of ANOVA demonstrated that feedstock, prepared temperature, and application proportion of biochar all have an influence on ES rate (Table 3 and Table 4). Specifically, PT (prepared temperature) showed a strong significant relationship with ES rate under pyrochar application (Table 3), indicating that a higher PT corresponded to a higher ES rate. Application proportion did not affect ES rate under pyrochar application at first, but there was a significant effect at R75. The combined effect of feedstock and application proportion significantly influenced the ES rate under hydrochar application (Table 4).

Further investigation of the relationship between the properties of biochar and the ES rate using correlation analysis demonstrated that BDc and average pore diameter (APD) were significantly correlated with ES. BDc showed a significantly positive relationship with RA, R25, R50, and R75 (*p* < 0.05), while APD showed a similar correlation coefficient at 6% application as BDc, but not significant at 2%. However, SSA and total pore volume (TPV) did not show a significant relationship with ES compared with BDc and APD (Table 5). 

Due to the high porosity and large SSA [27], pyrochar application improved soil aggregation by binding to other soil constituents [21]. Therefore, pyrochar significantly improved soil water retention capacity [28]. However, some researches also showed that pyrochar application did not influence soil porosity by direct pore contribution, creation of accommodation pores, or improved aggregate stability [29]. In addition to the improvement on water retention, there are other factors that contribute to the effect of pyrochar application on ES. On the one hand, pyrochar application increased soil porosity and hydraulic conductivity, which enhanced ES. On the other hand, pyrochar showed a water adsorption capacity, which inhibited ES [19]. In the present study, SSA of biochar did not show a significant relationship with ES (Table 5), which indicated that the improved soil water retention by BDs probably precede that by SSA. The water adsorption capacity of biochar was negligible compared with ES affected by decreased BDs. 

The effect of biochar application on ES is also closely related to soil properties. Former researches showed that pyrochar was a good soil amendment for sandy soil by enhancement of water retention and inhibit ES [20]. Results of the present study further demonstrated that pyrochar was beneficial to clayey soil (Figure 1 and Figure 2). Moreover, hydrochar, as a new type of biochar, also inhibited ES in clayey soil, although the inhibition was significantly lower than pyrochar (Figure 2). Nevertheless, whether the results of the present study on clayey soil could be extrapolated to other soils, such as sandy soil, is still unclear. Even if biochar application inhibited ES for both sandy and clayey soils, their mechanism should be different. The combined effects of biochar properties and soil properties on ES still need to be further investigated.

### 4.2. Biochar for Greenhouse Cultivation

Biochar has been intensively used in field crop production and proved to increase crop productivity [30,31]. As greenhouse cultivation increased, studies on biochar application under greenhouse systems have been conducted [32,33]. De Tender et al. (2016) applied biochar for greenhouse strawberry cultivation, and the result showed that 3% biochar application increased the fresh and dry weight of strawberry. To date, researches have shown that biochar application in greenhouses could increase yield [32], but most of them focused on the nutrient use efficiency improvement and disease resistance enhancement [33,34]. The effect of biochar application on water use efficiency improvement under greenhouse systems was still limited studied to date. 

Results of our study showed that biochar (especially pyrochar) application could inhibit ES. This indicated that biochar application had the potential for saving irrigation and increasing water use efficiency. Under practical greenhouse cultivation, polyethylene film is always used as a vapor barrier to preserve soil water content [35]. However, polyethylene film would increase cultivation cost and further induce soil degradation [36]. In this condition, biochar application was more efficient and environmentally friendly. However, some researchers thought biochar amendments could not improve the bioavailability of water and nutrients for all soils [28]. Further experiments should be conducted to investigate the optimal biochar type and application proportion for greenhouse cultivation.

## 5. Conclusions

The result of the present study showed that both pyrochar and hydrochar application to clayey soil reduced bulk density of soil and inhibited soil evaporation compared with CK. Soil evaporation inhibition of pyrochar is significantly better compared with that of hydrochar. The soil evaporation inhibition of biochars was closely related to the bulk density of biochar, rather than other biochar properties. The result of the present study indicated biochar, especially pyrochar application, could save irrigation by inhibiting soil evaporation in clayey soil, which showed a great potential for water conservation and water use efficiency improvement under greenhouse cultivation.

## Figures and Tables

**Table 1:** Basic properties of pyrochar and hydrochar.

Biochar Type	BDc (g/cm^3^)	SSA (m^2^/g)	APD (nm)	TPV (cm^3^/g)
SP500	0.12	20.73	6.41	0.033
WP500	0.10	22.38	5.08	0.028
SP700	0.11	114.20	5.02	0.143
WP700	0.11	32.03	4.25	0.034
SH260	0.26	1.44	17.93	0.006
WH260	0.36	0.30	17.07	0.001

BDc, SSA, APD, and TPV represent bulk density, specific surface area, average pore diameter, total pore volume of biochar, respectively. SP, WP, SH and WH represent sawdust pyrochar, wheat straw pyrochar, sawdust hydrochar and wheat straw hydrochar, respectively.

**Table 2:** Parameters of the fitting equation of soil evaporation curves under pyrochar and hydrochar application.

Treatments	Parameters of Linear Equation			Parameters of First-Order Kinetics Equation		
	a	b	R^2^	N_0_	b	R^2^
SP500-2%	0.874	9.687	0.931	139.149	0.012	0.981
SP500-6%	0.841	10.768	0.941	132.116	0.012	0.992
WP500-2%	0.854	11.847	0.923	128.802	0.013	0.986
WP500-6%	0.813	9.372	0.952	132.436	0.011	0.994
SP700-2%	0.853	13.366	0.912	125.467	0.015	0.984
SP700-6%	0.833	12.369	0.928	124.604	0.014	0.992
WP700-2%	0.832	15.372	0.898	118.735	0.017	0.983
WP700-6%	0.785	15.340	0.919	112.694	0.017	0.998
SH260-2%	0.838	15.169	0.904	119.914	0.016	0.989
SH260-6%	0.802	19.203	0.888	111.580	0.020	0.996
WH260-2%	0.793	22.023	0.850	108.855	0.023	0.992
WH260-6%	0.811	18.631	0.888	113.004	0.020	0.994
CK	0.787	23.937	0.816	107.718	0.025	0.986

**Table 3:** Soil evaporation rate under treatments of four pyrochar (SPC500, SPC700, WPC500, and WPC700) and two application proportion (2% and 6%).

Feedstock	PT(°C)	Application Proportion (%)	RA (%/h)	R25 (%/h)	R50 (%/h)	R75 (%/h)
CK			0.025 ± 0.003	1.84 ± 0.20	1.83 ± 0.20	1.78 ± 0.23
Saw dust	500	2%	0.012 ± 0.002	1.17 ± 0.13	1.19 ± 0.08	1.24 ± 0.05
		6%	0.012 ± 0.002	1.25 ± 0.09	1.22 ± 0.06	1.17 ± 0.05
	700	2%	0.015 ± 0.003	1.35 ± 0.20	1.34 ± 0.16	1.31 ± 0.07
		6%	0.014 ± 0.001	1.27 ± 0.07	1.31 ± 0.05	1.21 ± 0.05
Wheat straw	500	2%	0.014 ± 0.002	1.26 ± 0.12	1.27 ± 0.07	1.26 ± 0.03
		6%	0.011 ± 0.001	1.14 ± 0.08	1.16 ± 0.04	1.06 ± 0.01
	700	2%	0.017 ± 0.004	1.44 ± 0.26	1.44 ± 0.20	1.34 ± 0.11
		6%	0.017 ± 0.001	1.48 ± 0.05	1.42 ± 0.04	1.18 ± 0.02
		Feedstock (F)	ns	ns	ns	ns
		PT (T)	***	***	***	***
		Application proportion (P)	ns	ns	ns	***
		F × T	ns	ns	ns	ns
		F × P	ns	ns	ns	ns
		T × P	ns	ns	ns	ns
		F × T × P	ns	ns	ns	ns

PT represents prepared temperature of biochar. Mean value ± standard deviation. LSD (least significant difference) followed by *, **, ***, significance at 5%, 1%, and 0.1%, respectively; ns, non-significant. RA represents the average ES rate. R25, R50, and R75 represent ES rate when water loss reached 25%, 50%, and 75% of the total water.

**Table 4:** Soil evaporation rate under treatments of two hydrochar (SHC260 and WHC260) and two application proportion (2% and 6%).

Feedstock	PT(°C)	Application Proportion (%)	RA (%/h)	R25 (%/h)	R50 (%/h)	R75 (%/h)
CK			0.025 ± 0.003	1.84 ± 0.20	1.83 ± 0.20	1.78 ± 0.23
Saw dust	260	2%	0.016 ± 0.003	1.43 ± 0.17	1.43 ± 0.13	1.33 ± 0.07
		6%	0.020 ± 0.002	1.71 ± 0.10	1.63 ± 0.10	1.39 ± 0.06
Wheat straw	260	2%	0.023 ± 0.003	1.80 ± 0.13	1.76 ± 0.14	1.56 ± 0.10
		6%	0.020 ± 0.002	1.65 ± 0.14	1.60 ± 0.12	1.40 ± 0.07
		Feedstock (F)	ns	ns	ns	*
		Application proportion (P)	ns	ns	ns	ns
		F×P	*	*	*	*

PT represents prepared temperature of biochar. Mean value ± standard deviation. LSD (least significant difference) followed by *, **, ***, significance at 5%, 1%, and 0.1%, respectively; ns, non-significant. RA represents the average ES rate. R25, R50, and R75 represent ES rate when water loss reached 25%, 50%, and 75% of the total water.

**Table 5:** Correlation coefficients between biochar properties and soil evaporation rate.

2%	RA	R25	R50	R75	6%	RA	R25	R50	R75
PT	−0.460	−0.450	−0.473	−0.481	PT	−0.528	−0.586	−0.574	−0.673
BDc	0.850 *	0.849 *	0.858 *	0.861 *	BDc	0.815 *	0.825 *	0.835 *	0.908 *
SSA	−0.322	−0.303	−0.320	−0.307	SSA	−0.399	−0.481	−0.389	−0.343
APD	0.665	0.663	0.676	0.649	APD	0.821 *	0.847 *	0.859 *	0.920 **
TPV	−0.355	−0.335	−0.353	−0.332	TPV	−0.415	−0.493	−0.400	−0.331

PT, BDc, SSA, APD, and TPV represent prepared temperature, bulk density, specific surface area, average pore diameter, total pore volume of biochar, respectively. RA represents the average ES rate. R25, R50, and R75 represent ES rate when water loss reached 25%, 50%, and 75% of the total water. LSD (least significant difference) followed by *, **, ***, significance at 5%, 1%, and 0.1%, respectively.
